# Current and Emerging Molecular Therapies for Head and Neck Squamous Cell Carcinoma

**DOI:** 10.3390/cancers13215471

**Published:** 2021-10-30

**Authors:** Farzaneh Kordbacheh, Camile S. Farah

**Affiliations:** 1 Broad Institute of MIT and Harvard, Boston, MA 02142, USA; fkordbacheh@broadinstitute.org; 2 Department of Medical Oncology, Dana-Farber Cancer Institute, Boston, MA 02215, USA; 3 ACRF Department of Cancer Biology and Therapeutics, The John Curtin School of Medical Research, Australian National University, Canberra, ACT 0200, Australia; 4 The Australian Centre for Oral Oncology Research & Education, Nedlands, WA 6009, Australia; 5 Genomics for Life, Milton, QLD 4064, Australia; 6 Anatomical Pathology, Australian Clinical Labs, Subiaco, WA 6009, Australia; 7 Head and Neck Cancer Signalling Laboratory, Peter MacCallum Cancer Centre, Melbourne, VIC 3000, Australia

**Keywords:** head and neck oncology, head and neck squamous cell carcinoma, precision medicine, molecular therapies, targeted therapies, clinical trials, genomics

## Abstract

**Simple Summary:**

Next-generation sequencing of head and neck squamous cell carcinoma has revealed multiple new gene mutations, while simultaneously confirming the role of others in head and neck cancer tumorigenesis. The ever-expanding number of actionable druggable targets has fuelled a plethora of clinical trials assessing various pharmacotherapeutics in the management of this group of cancers. This review paper summarizes the state of play of molecular therapies used in head and neck oncology with particular focus dedicated to current FDA-approved drugs and emerging therapies undergoing advanced clinical trials.

**Abstract:**

Head and neck cancer affects nearly 750,000 patients, with more than 300,000 deaths annually. Advances in first line surgical treatment have improved survival rates marginally particularly in developed countries, however survival rates for aggressive locally advanced head and neck cancer are still poor. Recurrent and metastatic disease remains a significant problem for patients and the health system. As our knowledge of the genomic landscape of the head and neck cancers continues to expand, there are promising developments occurring in molecular therapies available for advanced or recalcitrant disease. The concept of precision medicine is underpinned by our ability to accurately sequence tumour samples to best understand individual patient genomic variations and to tailor targeted therapy for them based on such molecular profiling. Not only is their purported response to therapy a factor of their genomic variation, but so is their inclusion in biomarker-driven personalised medicine therapeutic trials. With the ever-expanding number of molecular druggable targets explored through advances in next generation sequencing, the number of clinical trials assessing these targets has significantly increased over recent years. Although some trials are focussed on first-line therapeutic approaches, a greater majority are focussed on locally advanced, recurrent or metastatic disease. Similarly, although single agent monotherapy has been found effective in some cases, it is the combination of drugs targeting different signalling pathways that seem to be more beneficial to patients. This paper outlines current and emerging molecular therapies for head and neck cancer, and updates readers on outcomes of the most pertinent clinical trials in this area while also summarising ongoing efforts to bring more molecular therapies into clinical practice.

## 1. Introduction

The druggable genome is defined as the altered genes or gene products that can interact with molecules exhibiting therapeutic properties [1,2]. Genomic alterations include gene deletions and amplifications that change gene abundance and their downstream products, alternative splicing or translocations that can create new proteins, and mutations such as single base changes that can modify protein activity [3,4]. Although genomic mutations are found in all cancers, they can be divided into two main groups. “Passenger” mutations, are the product of carcinogens and genomic instability that appear not to be involved in cancer progression, and “driver” mutations which are known to contribute to tumour development or progression [5,6,7]. The distinction between these mutations can change throughout the course of disease [8]. Driver mutations and their associated cellular pathways are known as “actionable” targets and may have significant diagnostic, prognostic, or therapeutic implications. A subset of these events may be druggable, making them valuable targets for therapeutic development and intervention [7,9].

Partial cancer genome datasets such as The Cancer Genome Atlas (TCGA, http://cancergenome.nih.gov) and the International Cancer Genome Consortium (ICGC, http://www.icgc.org) are available for many types of cancers [10,11]. TCGA data has more recently been used in large scale analysis of driver genes, incorporating over 9000 tumour exomes and resulting in identification of 299 unique cancer genes. This includes 59 novel driver genes and a number of known genes not previously associated with a specific tissue [12]. This comprehensive analysis across 33 cancer types highlighted the prevalence of clinically actionable cancer driver events in TCGA tumour samples [12]. It is estimated that nearly half the samples studied harboured a clinically relevant mutation, by predicting either sensitivity or resistance to certain treatments or clinical trial eligibility [12], with 57% of TCGA cases harbouring at least one potentially clinically actionable target.

Studies of whole-genome analysis of breast cancer patients has demonstrated new copy-number variations, descriptions of driver and other mutations, and elevated mutation rates in treatment-resistant tumours [13]. Banerji et al. has shown recurrent somatic mutations in *PIK3CA, TP53, AKT1, GATA3* and *MAP3K1* genes, and new recurrent mutations and deletions for *CBFB* and *RUNX1* [14]. Furthermore, studies have also shown that only 36% of mutations are detected as transcribed [15], and that many mutations encode truncated proteins [16]. It is unlikely that these novel findings would have been discovered using conventional sequencing or genotyping approaches.

Some actionable mutations are commonly altered in several different cancer types, and it is reasonable to assume that a therapy effective for one may be applicable in another. *BRAF V600E* is a gene marker for vemurafenib therapy in melanoma [17], which can also be used in ovarian cancer [18]. On the other hand, a specific genetic abnormality may not confer the same sensitivity to an agent across all cancers. Trastuzumab (Herceptin—anti HER2 monoclonal antibody mAb) has been shown to benefit breast and gastric cancer patients with HER2-amplification, but not in patients with endometrial or ovarian cancer [19,20,21].

PanCancer analysis of driver genes in TCGA studies has grouped head and neck squamous cell carcinoma (HNSCC) with other cancers of squamous origin, where they displayed a higher proportion of mutations in immune and receptor tyrosine kinase signalling genes as well as genes involved in chromatin remodelling [12]. *KIF1A*, a kinesin, was identified as a novel HNSCC driver gene. Interestingly though, this analysis identified that 71% of the 502 HNSCC samples in the dataset had potentially actionable mutations (either SNPs/Indels, CNVs or all), but that only 16% had a druggable mutation at any stage of development from preclinical to U.S. Food and Drug Administration (FDA)-approved at time of publication. This is in contrast to cutaneous melanoma, for which 93% of mutations were potentially actionable, and 90% were druggable, including a high proportion already FDA-approved. The availability of these large cancer datasets has resulted in the validation of many new driver mutations, clinically useful biomarkers and pharmacotherapeutic developments. Those with specific application in the treatment of HNSCC are listed in Table 1.

This paper summarises current and emerging molecular therapies in head and neck oncology driven by our understanding of gene mutations and genomic oncological pathways relevant to head and neck cancers. FDA-approved therapies, in addition to those in Phase II and III clinical trials, are outlined. Some such as EGFR inhibitors are more common place and used widely, while others such as p53 stimulants utilising adenovirus vectors are more recent advancements in the field and have received limited exposure. The generally poor outcomes for head and neck cancers continue to drive innovation in targeted therapeutic approaches, repurposing of currently available pharmacotherapeutics, and clinical trials to test safety, efficacy and potential combination therapies. Understanding of these current and emerging therapies for HNSCC necessitates a sound understanding of the signaling pathways involved in these cancers. For this, readers are directed to earlier papers detailing the molecular landscape, oncological pathways and druggable targets for head and neck cancers [22,23]. This review is intended to act as a coherent comprehensive summary of current and emerging molecular therapies for head and neck cancers (HNC). It outlines clinical trials (with corresponding ClinicalTrials.gov Identifiers) for HNC when available, and for other cancers where relevant in order to highlight clinical utility for possible extrapolation of relevant findings such as progression free survival (PFS), overall survival (OS), toxicity and adverse effects. Trials with reported outcomes in the literature are addressed in more detail than those still ongoing or terminated.

## 2. Current and Emerging Molecular Therapies

### 2.1. EGFR Pathway

Mutation and overexpression of *EGFR* is associated with a number of tumours including breast, lung, colorectal, ovary and prostate [24,25,26]. The frequency of EGFR mutations is 4% in HNSCC. Notably they have been the most promising candidate for developing molecular therapies.

EGFR is a transmembrane receptor of the human epidermal receptor (HER) family of growth factor receptors. Formation of EGFR homodimers or heterodimers (i.e., with HER2) trigger intracellular pathways that lead to cancer cell proliferation, apoptotic arrest, activation of invasion and metastasis, and stimulation of tumour-induced neovascularisation [27,28].

Currently, two primary approaches have been taken to target EGFR with different mechanisms including inhibition of tyrosine kinase domain (intracellular) activity with small molecules, and inhibition of extracellular ligand binding using monoclonal antibodies (mAbs). Anti-EGFR monoclonal antibodies (cetuximab, panitumumab) recognise EGFR exclusively and bind to its extracellular domain, compete for receptor binding and block ligand-induced EGFR tyrosine kinase activation [29]. EGFR tyrosine kinase inhibitors (erlotinib, gefitinib) compete in a reversible fashion with ATP and bind to the intracellular domain of EGFR tyrosine kinase thus inhibiting EGFR autophosphorylation and downstream signalling. EGFR tyrosine kinase inhibitors can also block different growth factor receptor tyrosine kinases such as VEGFR. Cetuximab is FDA- and EMEA-approved, in combination with radiotherapy, to treat locally advanced, unresectable HNSCC. It is also approved as monotherapy for metastatic disease in patients who have failed to respond to chemotherapy.

An open-label phase II randomised trial applied cetuximab, 5-fluorouracil and cisplatin with or without docetaxel in patients with recurrent and/or metastatic HNSCC. The median follow-up was 2 years. The median overall survival for the group with docetaxel (DPFC) compared to that without (PFC), was 8.9 vs. 10.6 months and response rates were 38.2% vs. 31.9%, respectively [30].

Binding of EGFR to its ligands triggers two key signalling pathways, PI3K-AKT and MAPK/ERK. These signalling pathways can be initiated by mutations in intermediate molecules. Mellinghoff et al. showed that up to 20% of glioblastoma patients were responsive to EGFR kinase inhibition which was associated with coexpression of EGFR vIII and PTEN [25]. Additionally, activating mutations in *KRAS* results in EGFR-independent signal activation and has been noted in up to 30% and 45% of patients with small-cell lung cancer and colorectal cancer, respectively, with a history of smoking [31,32]. Such patients show resistance or limited efficacy to cetuximab and panitumumab therapy [33,34]. MET amplification also leads to EGFR-independent activation of the PI3K-AKT pathway through activation of HER3. Inhibition of MET signalling has been shown to restore the sensitivity of lung cancer cell lines to gefitinib [35].

#### 2.1.1. Irreversible EGFR Inhibitors

In HNSCC, tyrosine kinase inhibitors (TKIs) are generally quinazoline-derived, low molecular weight synthetic molecules that competitively block the intracellular ATP binding domain of EGFR and consequently inhibit the activation and phosphorylation of EGFR and its downstream cellular intermediates [36,37]. These molecules can be administrated orally. Gefitinib and erlotinib are relatively specific for EGFR and are currently being used for lung cancer [37,38].

Gefitinib (Iressa, ZD1839, NSC 715055) is the first TKI to reach Phase III trials in HNSCC but according to several trial study failures it has been withdrawn in the United States. In a Phase III study (NCT00206219), 477 patients with recurrent or metastatic HNSCC were randomly assigned to compare survival rate after treating with gefitinib 250 mg/day (FDA-approved dose for advanced non-small-cell lung cancer (NSCLC)) or 500 mg/day (orally) or standard methotrexate (40 mg/m^2^ intravenously). The results showed that neither gefitinib 250 nor 500 mg/day improved overall survival compared with methotrexate [39]. Another Phase III trial conducted by the Eastern Cooperative Oncology Group (ECOG) with 270 patients was closed early at interim analysis due to the unlikelihood of meeting its endpoint goals. The results showed that adding gefitinib to docetaxel was well tolerated but did not improve outcomes in poor prognosis of patients with recurrent or metastatic HNSCC (NCT00088907) [40]. Therefore, clinical development of gefitinib for HNSCC is unlikely to proceed further.

Erlotinib (Tarceva, OSI-774) is an approved therapy in the U.S. for advanced pancreatic cancer [41] and NSCLC [42], and so far has demonstrated promising results in HNSCC clinical trials [43]. In a multicentre Phase II study, 115 patients were enrolled to determine the efficacy and safety profile of erlotinib as a single agent in advanced recurrent and/or metastatic HNSCC. Almost half of patients (46%) were treated with constant 150 mg/day and had a partial response rate of 4.3%. In addition, approximately one third of patients (38%) had disease stabilisation for a median duration of 16.1 weeks which was comparable with palliative chemotherapy [44]. In a Phase I/II study, the effect of erlotinib was assessed in combination with cisplatin to target patients with recurrent or metastatic HNSCC (NCT00030576). Fifty-one patients were treated with erlotinib 100 mg/day (orally) and cisplatin 75 mg/m^2^ (intravenously) every 21 days. The overall response rate was 21%, and disease stabilisation was observed for 49% of patients. Median PFS and median OS were 3.3 and 7.9 months, respectively. These results are comparable with a Phase III trial of cetuximab plus cisplatin [45,46].

In another Phase II trial, the effect of adding erlotinib to cisplatin and radiotherapy was investigated in 204 patients with locally advanced HNSCC. In this study, erlotinib (150 mg/day) did not elevate the toxicity of cisplatin and radiotherapy, however no significant increase was observed for complete response rate (CRR) or PFS [47]. A Phase II study of erlotinib as adjuvant treatment for locally advanced HNSCC (NCT01515137) significantly decreased proliferation in HNSCC, with additional effect from the nonsteroidal anti-inflammatory drug sulindac [48]. However, erlotinib therapy for patients with incurable cutaneous SCC in a single-arm Phase II clinical trial showed an overall response rate of 10% and a disease control rate (partial response + stable disease) of 72% [49]. In a recent study, HNSCC tumours collected from patients with MAPK1p.E322K mutation and treated with EGFR inhibitor, conferred heightened sensitivity to erlotinib in vivo and proceeding to patient recruitment in a clinical trial [43]. Its combination with other drugs such as docetaxel and cisplatin as an induction chemotherapy in loco-regional advanced HNC (Phase II, NCT00935961), or carboplatin and cetuximab in advanced HNC (Phase II, NCT01283334) [50] have been investigated.

In addition to gefitinib and erlotinib which are specific EGFR TKIs, lapatinib and vandetanib possess dual specificity for EGFR/HER2 and EGFR/VGFR2, respectively. Lapatinib (Tykerb) (GW572016) is FDA-approved for use in HER2-positive breast cancer in combination with capecitabine (Xeloda). In 2010, lapatinib received approval for the treatment with hormone receptor positive metastatic breast cancer in postmenopausal women with overexpression of HER2 receptor and for whom hormonal therapy was indicated (in combination with letrozole). In a multi-institutional Phase II study, 45 recurrent and/or metastatic HNSCC patients were recruited to determine efficacy and safety of lapatinib as a single agent (NCT00098631). Although lapatinib was well tolerated by HNSCC patients, it appeared to be inactive in EGFR-inhibitor naive or refractory subjects [51]. In a study of 44 patients with recurrent or metastatic HNSCC (NCT01044433), the combination of capecitabine and lapatinib as first-line therapy showed an overall response rate of 25% (90% CI, 15–38%) with a median OS of 10.7 months [52]. Addition of lapatinib to chemoradiotherapy and its use as long-term maintenance therapy (NCT00424255) in 688 patients (lapatinib, *n* = 346; placebo, *n* = 342) did not offer any efficacy benefits with a median follow-up time of 35.3 months, but had additional toxicity compared with placebo in patients with surgically treated high-risk HNSCC which resulted in the study being terminated early [53].

Vandetanib (ZD6474) is an orally bioavailable EGFR and VGFR-2 tyrosine kinase inhibitor. So far, it has been shown to effectively restore the sensitivity of HNSCC models to cisplatin and radiation in in vitro and in vivo studies [54]. It is important to note that trials for locally advanced HNSCC incorporating EGFR inhibition (cetuximab, erlotinib, panitumumab) with chemoradiation failed to show an advantage in PFS or OS over chemoradiation alone [55].

#### 2.1.2. Anti-EGFR Monoclonal Antibodies

There are several anti-EGFR monoclonal antibodies developed for targeting human epithelial cancers of which at least five have been evaluated in clinical trials as potential EGFR-targeted therapy for HNSCC patients [36]. The initial concept for developing these therapies was that they would block receptor ligand interactions such as EGFR to EGF or TGF-α and consequently inhibit downstream signal transduction. However, the response to the antibody-EGFR interaction is complex in nature and is thought to act via different mechanisms. It has been shown that both monoclonal human immunoglobulin G1, IgG1 (cetuximab, matuzumab and nimatuzumab) and IgG2 antibodies (panitumumab) induce antibody-dependent cellular cytotoxicity (ADCC) [56,57]. Additionally, antibody structural differences and their EGFR epitope targets may affect the efficacy and toxicity of the drug [58].

Cetuximab is a chimeric human-murine IgG1 monoclonal antibody (mAb) (derivative of mAb225, C225) and a potent inhibitor of EGFR activation and blocks phosphorylation and activation of receptor-associated kinases and consequently inhibits cell cycle progression and proliferation, angiogenesis and vascular formation (decreased matrix metalloproteinase and vascular endothelial growth factor production) and metastasis [56,59]. EGFR is constitutively expressed in many normal epithelial tissues, including skin and hair follicles. Overexpression of EGFR is also detected in colon and rectum cancers.

Cetuximab (Erbitux^®^) was first approved by FDA in 2004 when it showed clinically significant activity when administrated alone or in combination with irinotecan (Camptosar) in patients with irinotecan-refractory colorectal cancer [60]. In 2006, it was FDA-approved for treatment of loco-regionally advanced HNSCC in combination with high dose radiotherapy (NCT00004227). The Phase III multicentre study was designed to evaluate the effect of radiotherapy with or without cetuximab (initial dose of 400 mg/m^2^ followed by 250 mg/m^2^ weekly) in treating 424 patients suffering from Stage III or IV oropharynx, hypopharynx, or larynx cancers [61]. Treatment with cetuximab plus radiation therapy (RT) (211 patients) increased the overall survival median duration to 49 months compared to 29.3 months for patients who were treated with radiotherapy alone (213 patients) [62]. Although this study showed a very positive outcome, treatment resistance and treatment failure must be viewed in the context of other mutations (such as p53) involved in HNSCC. One of the ways to overcome this issue might be adding agents to current known treatments. For example, cisplatin (a FDA-approved drug which induces apoptosis) can be employed in patients with mutated/damaged DNA [63].

In 2006, FDA also approved cetuximab for use in combination with platinum-based chemotherapy and 5-fluorouracil as first-line treatment of recurrent/metastatic conditions when it significantly increased the median overall survival from 7.4 months to 10.1 months, and as a single agent in recurrent/metastatic disease after failure of platinum-based chemotherapy when it increased the median progression-free survival time from 3.3 to 5.6 months as well as the response rate from 20% to 36% (*p* < 0.001) (EXTREME; NCT00122460) [64]. It was also approved by European Medicines Agency (EMA) for HNSCC and colorectal targeted therapy. Numerous Phase I, II and III studies are being performed on the effect of cetuximab as a single agent or with combination of radiotherapy and chemotherapy and other agents to treat HNSCC, but some side effects such as skin rash, drug resistance and infusional allergic reactions due to murine Ig component have been associated with this drug [46,62,65,66,67,68,69]. The effect of radiotherapy plus cetuximab or cisplatin was assessed in 849 HPV-positive oropharyngeal cancer patients in a Phase III randomised, multicentre, noninferiority trial (NCT01302834). 425 received radiotherapy plus cetuximab and 424 received radiotherapy plus cisplatin. Radiotherapy plus cetuximab showed inferior OS and PFS compared with radiotherapy plus cisplatin. Estimated 5-year overall survival was 77.9% in the cetuximab group versus 84.6% in the cisplatin group. Progression-free survival was significantly lower in the cetuximab group compared with the cisplatin group (5-year proportions 67.3% vs. 78.4%), and locoregional failure was significantly higher in the cetuximab group compared with the cisplatin group (5-year proportions 17.3% vs. 9.9%) [70]. Therefore, the use of cetuximab not only requires a comparison against other available conventional methods with or without alternative drugs, but also requires an understanding of each patient’s clinical status, history and risk profile [71].

Panitumumab (Vectibix^®^) is a fully humanised mAb that does not stimulate ADCC as strongly as cetuximab, and has been associated with minimal infusion-related reactions and may also reduce the production of neutralising antibodies [72]. Panitumumab was approved by FDA in 2006 for EGFR-expressing metastatic colorectal cancer (CRC) with disease progression on or following fluoropyrimidine-, oxaliplatin- and irinotecan containing chemotherapy regimens [73]. The approval was as a result of randomised Phase III multinational study with 463 patients suffering from metastatic colorectal cancer receiving either best supportive care (BSC) alone (chemotherapy regimens containing a fluoropyrimidine, irinotecan, and oxaliplatin) (232 patients) or BSC plus panitumumab, 6 mg/kg intravenously (231 patients). Median and mean PFS for patients receiving panitumumab and those receiving only BSC were 56 and 96.4 days, and 51 and 59.7 days, respectively [73].

Currently, panitumumab is in Phase III trials for HNSCC. A multinational Phase III study was performed to evaluate the efficacy and safety of panitumumab chemotherapy combined with cisplatin and 5-fluorouracil as first-line treatment for 658 patients with recurrent and/or metastatic HNSCC (NCT00460265) [74]. Both median OS (11.1 vs. 9 months) and median PFS (8.8 vs. 4.6 months) improved in those treated with additional panitumumab (327 patients) compared to the control group (a regimen of cisplatin and fluorouracil in 330 patients), but the improvements were not significant. In another Phase III trial evaluating the effect of combination of standard fractionation RT with cisplatin or panitumumab in 320 patients with locally advanced stage III and IV HNSCC (NCT00820248), panitumumab plus accelerated-fractionation RT was not superior to cisplatin plus standard-fractionation. The 2-year PFS was 73% in the cisplatin group and 76% in the panitumumab group, and 2-year overall survival was 85% and 88% in cisplatin and panitumumab groups, respectively [75].

Like cetuximab, many active Phase I and II trials are being performed to investigate the efficacy and safety of panitumumab as first or second line treatment for HNSCC despite development of some side effects including skin rashes which remains the main issue associated with EGFR-targeted antibodies and has even being considered as a marker for drug efficiency [29,76].

There are other promising mAbs being evaluated in Phase III trials for HNSCC including zalutumumab (fully humanised IgG1) and nimotuzumab (humanised murine IgG1) that have been tested as single agent or combinational therapy. Zalutumumab showed reasonable efficacy in HNSCC patients [77], extending progression-free survival in patients with recurrent HNSCC who had failed platinum-based chemotherapy (NCT00382031) but did not increase overall survival [78]. Nimotuzumab has shown less skin toxicity compared to cetuximab [79] and is currently in a Phase III trial recruiting patients aimed at improving loco-regional control rate and overall survival of locally advanced HNSCC compared to concurrent chemo-radiotherapy (NCT00957086).

### 2.2. PI3K/AKT/mTOR Pathway

Alterations of PI3K pathway have been associated with many solid tumours, therefore it encompasses valuable therapeutic targets. So far, a number of therapeutic agents have been developed which target key molecules within the pathway including PI3K, AKT and mTOR. Moreover, there are also other targeted therapies targeting other intermediate proteins within the pathway. Some are approved for use while others are potential targeted therapeutics under investigation.

#### 2.2.1. PI3K Inhibitors

PI3K inhibitors in HNSCC are currently directly or indirectly (solid tumours) in clinical trial investigations. They can be divided into three major classes:

##### PI3K/mTOR Inhibitors

GDC-0980 is being investigated in Phase I and II trials for conditions including renal cell carcinoma (RCC) (NCT01442090), solid tumours (NCT01301716 and NCT01332604) and currently recruiting patients for prostate cancer (NCT01485861).

NVP-BEZ235 could reach Phase II clinical trials, however has been delayed due to cases being terminated (transitional cell carcinoma, NCT01856101 and metastatic, castration-resistant prostate cancer, NCT01717898) or withdrawn (advanced endometrial carcinoma, NCT01290406 and malignant PEComa, NCT01690871) in order to reformulate the drug.

A Phase II comparative study of NVP-BEZ235 and everolimus in patients with advanced pancreatic neuroendocrine tumours did not demonstrate increased efficacy compared with everolimus which might be due to a poorer tolerability profile. The median PFS was 8.2 months with BEZ235 versus 10.8 months with everolimus [80]. BGT226 has completed a Phase I/II study sponsored by Novartis for advanced solid malignancies including advanced breast cancer (NCT00600275).

##### Pan-Class I PI3K Inhibitors

Pan PI3K inhibitors include PX-866, BKM120 and SAR245408 (XL147).

PX-866 showed modest activity as a single agent therapy in patients with recurrent or metastatic prostate cancer [81]. It is mostly in active clinical Phase II trials in combination with drugs such as vemurafenib for advanced melanoma (NCT01616199), cetuximab for incurable progressive, recurrent or metastatic HNSCC (NCT01252628) and docetaxel for HNSCC (NCT01204099).

BKM120 is in Phase II trials as a single or combination therapy for many cancers including head and neck cancers. BKM120 monotherapy is being evaluated for advanced HNSCC (NCT01527877) and metastatic and recurrent or progressive HNC (NCT01737450). Trials assessing BKM120 in combination with cetuximab (NCT01816984) and cisplatin (NCT02113878) for recurrent or metastatic HNC are also active.

SAR245408 (XL147) has not been tested in HNC directly but has successfully completed Phase I and/or II trials in other conditions including breast cancer in combination with letrozole (NCT01082068), locally advanced or metastatic solid tumours in combination with oral MSC1936369B (NCT01357330), and advanced or recurrent endometrial cancer as monotherapy (NCT01013324).

##### PI3Kα (p110α) Inhibitors

The p110α protein (protein phosphatidylinositol 4,5-bisphosphate 3-kinase catalytic subunit alpha isoform) is a subunit of the PI3K protein that is encoded by *PIK3CA* (phosphatidylinositol-4,5-bisphosphate 3-kinase, catalytic subunit alpha). *PIK3CA* is one of the most commonly seen mutations in HNSCC (10–12%) alongside with *BRCA1* (6%), and *BRCA2* (7–9%) [82]. A comprehensive genomic study of HNSCC in 2015 showed 25% of mutated PIK3CA displayed concurrent amplification, with an additional 20% of tumours containing focal amplification without evidence of mutation. Seventy-three per cent of *PIK3CA* mutations localized to Glu542Lys, Glu545Lys and His1047Arg/Leu hotspots [83].

Alpelisib (BYL719) is an α-specific PI3K inhibitor, and was approved by the FDA in 2019 to treat certain types of breast cancer. Its combination with paclitaxel showed a challenging safety profile in patients with advanced solid tumours [84]. Currently, a Phase I trial of a combination of BYL719 with cetuximab and intensity-modulated radiation therapy is active for stage III/IVB HNSCC (NCT02282371).

#### 2.2.2. AKT Inhibitors

Perifosine is a lipid-based AKT inhibitor which interacts with pleckstrin homology domain of AKT and inhibits its binding to PIP_3_ [85]. It has been successfully tested in Phase I and II trial studies including recurrent/progressive malignant gliomas (Phase II, NCT00590954), recurrent pediatric solid tumours (Phase I, NCT00776867) and non-small -cell lung cancer (Phase II, NCT00399789). In HNSCC, it has shown antiproliferative activity in cell lines by blocking cell cycle progression at G1-S and G2-M in preclinical studies [86]. However, a Phase II clinical study on recurrent or metastatic HNC (NCT00062387) was terminated due to lack of antitumor activity as a single agent [87] and currently has no clinical trial for HNC.

MK2206 is another AKT inhibitor that has reached Phase II for progressive, recurrent, or metastatic adenoid cystic carcinoma of the oral cavity (NCT01604772), recurrent or metastatic HNC (NCT01349933) and recurrent nasopharyngeal carcinoma (NCT01370070). Other examples of AKT inhibitors in Phase I or II clinical studies on a variety of cancers are AZD5363, triciribine, AR-67 and GSK690693.

#### 2.2.3. mTOR Inhibitors

Rapamycin (Sirolimus, Rapamune^®^) received FDA approval in 1999 as an immuno-suppressant drug for the prophylaxis of organ rejection in patients receiving renal transplants. It binds to mTORC1 and consequently blocks the phosphorylation of its downstream substrates, S6K1 and eukaryotic initiation factor-4E (eIF-4E)-binding protein (4E-BP1) [88]. Due to its lipophilic structure [89] different formulations of rapamycin have been tested to improve its poor water solubility and bioavailability for clinical application. So far, three analogues of rapamycin have been developed; everolimus (RAD001, Afinitor^®^), temsirolimus (CCI-779, Torisel^®^) and deforolimus (ridaforolimus, AP23573). Although modified formulations of these drugs enhanced their bioavailability and half-life, as well as reducing their immunosuppressive effects compared to rapamycin, all target mTOR and result in arresting cell cycle at the G1 phase [90,91].

Everolimus is FDA-approved for five cancerous conditions including advanced RCC (2009), subependymal giant cell astrocytoma associated with tuberous sclerosis (2010), progressive neuroendocrine tumours of pancreatic origin (2011), advanced hormone receptor-positive HER2-negative breast cancer (2012), paediatric and adult patients with subependymal giant cell astrocytoma (2012), and neuroendocrine tumours of gastrointestinal or lung origin with unresectable, locally advanced or metastatic disease (2016). A Phase II trial (NCT00942734) assessing the combination of erlotinib and everolimus did not show significant benefit in unselected patients with platinum-resistant metastatic HNSCC despite an acceptable toxicity profile. Twelve-week PFS was 49%, median PFS 11.9 weeks, and median OS 10.25 months [92].

Temsirolimus was approved by the FDA in 2007 for treatment of advanced RCC [93]. Currently, in HNC patients it has reached Phase II clinical trials as monotherapy (NCT01172769) and combinational therapy with cetuximab (NCT01256385) and paclitaxel and carboplatin (NCT01016769) in recurrent and/or metastatic HNSCC. However, its Phase II trial study for patients with platinum-refractory/ineligible, advanced HNSCC in combination with erlotinib was terminated (NCT01009203) due to abrogation of the mTOR pathway [94].

It is noteworthy that one of the drawbacks of using mTOR inhibitors might be PI3K activation as a result of negative feedback response between phosphorylation of insulin receptor substrate protein 1 (IRS1) by S6K1 and blockage of insulin like growth factor 1 (IGF-1) signalling to PI3K [95]. Therefore, the search for agents with dual PI3K/mTOR inhibition activity might be a better approach for PI3K-pathway-targeted therapy.

### 2.3. RAS/RAF/MEK/MAPK Pathway

Mutations in RAS/RAF/MEK/ERK (MAPK) pathway are common in all cancer types including head and neck, breast and prostate to name a few. Targeted therapy has gained remarkable attention and many key protein inhibitors have been introduced including RAF and MEK1 and MEK2 inhibitors. Although RAS inhibitors may be of a great value in RAS pathway suppression, clinical trials have not achieved satisfactory results as the inhibitors could not appropriately affect protein targets. However, in 2013 data were presented suggesting that it may be feasible to target mutant RAS proteins directly or target other unique features of *RAS*-driven tumours. Here we give some examples of inhibition of two important downstream proteins of RAS pathway, including MEK and RAF.

#### 2.3.1. RAS Inhibitors

*RAS* (*KRAS*, *NRAS* and *HRAS*) is the most frequently mutated gene family in cancers. Up until 10 years ago, *RAS* was termed ’undruggable’; however, the success of allele-specific covalent inhibitors against the most frequently mutated version of *RAS* provided the opportunity to evaluate the best therapeutic strategies to treat *RAS*-driven [96]. Mutations in *HRAS* occur in 4–8% of patients with recurrent and/or metastatic HNSCC [83,97].

Tipifarnib (Zarnestra) is a potent and highly selective inhibitor of farnesyltransferase (FTase). FTase facilitates the attachment of farnesyl groups to signalling proteins that are required for cell membrane localization. All RAS isoforms are FTase substrates; however, only HRAS is exclusively dependent upon farnesylation and therefore can be targeted via tipifarnib-mediated inhibition of FTase [98]. Tipifarnib demonstrated promising efficacy in a phase II clinical trial in patients with recurrent and/or metastatic HNSCC with HRAS mutations for whom limited therapeutic options exist (NCT02383927). Objective response rate for evaluable patients with high-VAF HNSCC was 55%. Median PFS on tipifarnib was 5.6 months versus 3.6 months on last prior therapy and median OS was 15.4 months. The most frequent adverse events among the 30 patients with HNSCC were anaemia (37%) and lymphopenia (13%) [97]. Currently a Phase II clinical trial is recruiting patients to assess the safety and efficacy of tipifarnib in HNSCC with *HRAS* mutations and impact of *HRAS* on response to therapy (AIM-HN/SEQ-HN) (NCT03719690).

#### 2.3.2. RAF Inhibitors

As mutations in *CRAF* (*RAF1*) are extremely rare, whereas *BRAF* mutations are profoundly oncogenic, BRAF protein kinases have been targeted by a number of BRAF inhibitors including sorafenib, vemurafenib and dabrafenib.

Sorafenib (tosylate salt of BAY 43-9006, Nexavar^®^) is a small molecule which inhibits RAF kinase and VEGF receptor kinase. It has been used for Ras gene mutated tumours and tumours overexpressing growth factor receptors in the Ras/Raf/Mek pathway, and inhibiting tumour angiogenesis or neovascularization via inhibition of VEGFR-2, VEGFR-3, and/or PDGFR-β including advanced clear cell renal carcinoma and hepatocellular carcinoma as well as hepatocellular carcinoma [99]. Sorafenib was approved by FDA for the treatment of patients with advanced RCC in 2005, for the treatment of unresectable hepatocellular carcinoma in 2007, and for the treatment of locally recurrent or metastatic progressive differentiated thyroid carcinoma (DTC) in 2013 (http://www.cancer.gov/cancertopics/druginfo/fda-sorafenib-tosylate). It is currently in Phase II clinical studies for head and neck cancers. In 2007, the efficacy and safety of sorafenib was evaluated in 27 patients with recurrent and/or metastatic HNSCC and nasopharyngeal carcinoma (NPC) in a Phase II trial [100]. Five patients showed evidence of disruption of the EGFR-Ras-Raf-MEK-ERK signalling pathway, a proapoptotic effect, and less convincingly, an effect on angiogenic pathways [100]. Although, sorafenib was well tolerated by patients, it had a modest anticancer activity compared to other monotherapies for HNSCC in Phase II clinical trials [44,100,101,102,103]. Williamson et al. conducted a Phase II trial in 2010 to evaluate the efficacy and safety of sorafenib as a single agent (400 mg twice daily in 28-day cycles) in 41 patients with metastatic or recurrent HNSCC (NCT00096512) and achieved similar results to Elser et al. [99]. It is currently in active Phase II study in combination with carboplatin and paclitaxel to treat patients with HNSCC (NCT00494182), and has been assessed in a Phase II study in combination with cetuximab for recurrent or metastatic HNSCC and other types of oral and nasal cavity (NCT00939627).

Vemurafenib (Zelboraf^®^) was approved by FDA in 2011 for the treatment of unresectable or metastatic melanoma with the *BRAF V600E* mutation (http://www.cancer.gov/cancertopics/druginfo/fda-vemurafenib). It is currently being tested as monotherapy for locally advanced thyroid cancer (Phase II, NCT01709292), melanoma (Phase II, NCT01813214) and relapsed or refractory hairy cell leukaemia (Phase II, NCT01711632). It is also being evaluated in combination with other drugs such as everolimus or temsirolimus for advanced cancer and solid tumours (Phase I, NCT01596140), bevacizumab for stage IV BRAFV600 mutant metastatic melanoma (Phase II, NCT01495988) and cetuximab for advanced solid cancers (Phase I, NCT01787500). A Phase I study of vemurafenib plus HL-085 in solid tumours with BRAF V600 mutation is currently recruiting patients (NCT03781219).

In 2013, dabrafenib (Tafinlar^®^) was approved as a single agent for treatment of unresectable or metastatic melanoma with *BRAF V600E* mutation. In 2014 it was approved for use in combination with trametinib (MEK1/2 inhibitor) which was also FDA-approved in 2013 to treat patients with unresectable or metastatic melanoma with a BRAF V600E/K mutation. The combination of dabrafenib/trametinib received FDA approval in 2018 as an adjuvant treatment for *BRAF V600E*-mutated, stage III melanoma after surgical resection. This was based on the results of the COMBI-AD Phase III study making it the first oral chemotherapy regimen to prevent cancer relapse for node positive, *BRAF*-mutated melanoma. Like vemurafenib, it is currently recruiting patients for Phase II studies including use of dabrafenib with stereotactic radiosurgery in BRAF V600E melanoma brain metastases (NCT01721603), with or without trametinib for recurrent thyroid cancer (NCT01723202) and with trametinib and navitoclax for solid tumours which are metastatic or cannot be removed by surgery (NCT01989585).

#### 2.3.3. MEK Inhibitors

In contrast to RAF and MEK1/2 which have narrow substrate specificities, activated ERK1/2 catalyse the phosphorylation of multiple cytoplasmic and nuclear substrates, and regulate diverse cellular responses such as mitosis, embryogenesis, cell differentiation, motility, metabolism, angiogenesis, and programmed cell death [104]. MEK1/2 represents a bottleneck in the activation of diverse cellular responses, many of which are of significantly important in tumorigenesis [105,106].

CI-1040 was the first MEK inhibitor to reach Phase I trials in 2000 [107], and since then many of its analogues have reached clinical trial studies. To date, trametinib is the only FDA-approved MEK inhibitor targeted therapy as the other agents either showed only limited efficiency as a single agent or failed to show satisfactory results in the examined tumour types [105]. Some examples of new and emerging MEK inhibitors are pimasertib, refametinib, selumetinib and MEK162.

Despite ongoing studies of MEK inhibitors, only a small group of cancers can be targeted by these molecules, including HNSCC. This can be improved by detection of biomarkers contributing to therapeutic responsiveness. So far, only *NRAS* and *BRAF V600* mutations have shown a consistent correlation with MEK inhibitors [105].

Trametinib (Mekinist^®^, GSK1120212) is an orally bioavailable, allosteric and selective inhibitor of MEK1/2 enzymes. The FDA approved trametinib for the treatment of patients with unresectable or metastatic melanoma with BRAF V600E/K mutations as a single agent in 2013 and in combination with dabrafenib (Tafinlar^®^) in 2014. In 2018, the combination of dabrafenib/trametinib was approved by the FDA as adjuvant treatment for *BRAF V600E*-mutated, stage III melanoma after surgical resection [108].

Trametinib (Mekinist^®^) is indicated as monotherapy or combinational therapy for the treatment of patients with unresectable or metastatic melanoma with a *BRAF V600* mutation.

Trametinib plus dabrafenib significantly prolonged PFS and OS, improved objective response rates and preserved health-related quality of life to a greater extent than dabrafenib (in the double-blind COMBI-d study) and vemurafenib (in the open-label COMBI-v study) in two large, randomized, Phase III studies in treatment-naïve patients with unresectable or metastatic melanoma with *BRAF* (*V600E/K*) mutation [109]. It is currently recruiting patients for Phase I, II and III trials as mono or combinational therapy.

Selumetinib (AZD6244, ARRY-142886) is a new potent MEK1/2 inhibitor and, like other MEK inhibitors, acts via the ATP noncompetitive mechanism and therefore has no significant inhibitory effect on other serine/threonine kinases [110]. In 2020, selumetinib received FDA approval for the treatment of children with neurofibromatosis type 1 (NF1) based on a clinical trial (NCT01362803). Selumetinib is now being tested for different cancer types including thyroid cancer [111] (NCT01843062), ovarian cancer, peritoneal cancer (NCT00551070), and advanced solid malignancies with cixutumumab combination (NCT01061749).

There are other MEK inhibitors which inhibit solid tumours such as cobimetinib in combination with pictilisib (PI3K inhibitor) in locally advanced or metastatic solid tumours (Phase I, NCT00996892), pimasertib and SAR245409 (PI3K/mTOR inhibitor) for locally advanced or metastatic solid tumours (Phase I, NCT01390818), and MEK162 and RAF265 (RAF inhibitor) in advanced solid tumours harbouring RAS or BRAF V600E mutations (Phase I, NCT01352273).

### 2.4. NOTCH Pathway

As the NOTCH pathway has dual oncogenic and tumour suppressor actions, therapeutically targeting this pathway is associated with some concern. To date, a variety of γ-secretase inhibitors (GSI) are being evaluated in preclinical and clinical trials to target NOTCH1 by inhibiting NICD cleavage and consequently its translocation into the nucleus [112,113]. Although there are no clinical trials currently being performed in HNSCC, there are studies targeting different tumour types including melanoma [114] and solid tumours [115]. Insights gleaned from these may inform therapeutic approaches in HNC, given the significance of the NOTCH pathway in HNSCC.

### 2.5. MET Pathway

MET regulation can be affected by overexpression, constitutive kinase activation, gene amplification, mutation or paracrine/autocrine activity via hepatocyte growth factor (HGF) [116,117]. The expression of MET can be targeted at RNA level by a novel approach of using small RNA molecules including small interfering RNA (siRNA) and micro RNA (miRNA) [118]. HGF competitors and antibodies are other therapeutic alternatives. NK4 is a member of N-terminal hairpin domain and Kringle domain (NK) inhibitor family which acts as a competitive antagonist for HGF. In vitro analysis has shown that NK4 inhibits angiogenesis induced by basic fibroblast growth factor (bFGF) and vascular endothelial growth factor (VEGF) as well as HGF [119]. AMG102 (rilotumumab) is a fully humanised monoclonal anti-HGF IgG which binds to β-chain of HGF and prevents its binding to MET. After positive results in preclinical and Phase I and II clinical studies on solid and lung tumours, it is currently recruiting patients for a Phase III trial for lung-MAP: S1400 biomarker-targeted second-line therapy in treating patients with recurrent stage IIIB-IV squamous cell lung cancer (NCT02154490).

Various MET inhibitors which act as kinase inhibitor at MET ATP binding sites, are currently under preclinical and clinical investigation. Tivantinib (ARQ197) is a MET kinase inhibitor which is being evaluated in different phases of clinical trials including investigating the effects of tivantinib plus erlotinib versus single agent chemotherapy in locally advanced or metastatic NSCLC (Phase II, NCT01395758), tivantinib in treating younger patients with relapsed or refractory solid tumours (Phase I, NCT01725191) and tivantinib in combination with chemotherapy in metastatic colorectal cancer (Phase I/II, NCT01075048). Patients are also being recruited for a Phase II randomized study to investigate the effects of cetuximab with or without tivantinib in HNC which is recurrent, metastatic, or cannot be removed by surgery (NCT01696955).

Ficlatuzumab is a humanised monoclonal antibody currently in a Phase II trial (NCT03422536), having been assessed in a Phase I trial assessing a combinational therapy with cetuximab in patients for recurrent/metastatic HNSCC which was completed in 2019 (NCT02277197). Results are not currently available for this study.

Foretinib is a c-Met receptor and vascular endothelial growth factor receptor 2 (VEGFR-2) inhibitor. A Phase II trial of single-agent foretinib (GSK1363089) in patients with recurrent or metastatic HNSCC (NCT00725764) was well tolerated and showed prolonged disease stabilisation [120].

Some other MET inhibitors that are currently in clinical investigation are crizotinib, exelixis (XL184) and MGCD265. The Phase I clinical trial of SGX523 to treat solid tumours (NCT00606879) however was terminated due to unexpected renal toxicity [121]. Moreover, there are other characterised small molecules including SU11274 [122] and PHA665752 [123] which so far have shown promising results in in vitro and in vivo studies.

### 2.6. JAK/STAT Pathway

The role of Janus kinase (JAK) and STAT pathway has been extensively studied in cancers including breast, lung, and head and neck (HNSCC). Activation of JAK leads to phosphorylation of STAT proteins, resulting in dimerization and translocation into the nucleus where they act as transcription factors with pleiotropic downstream effects. Activation of STAT3 and STAT5 results in elevated cell proliferation and survival, angiogenesis, and immune evasion [124].

Many molecules affecting STAT3, STAT5 and JAKs, particularly JAK2, are in preclinical and clinical studies. For example, it has been shown that combining radiotherapy with MEK1/2, STAT5 or STAT6 inhibition could reduce survival of HNC cell lines [125]. Therapies which inhibit STAT (mostly STAT3) are being researched in vitro and in vivo, however not many of these agents have reached clinical development. A novel STAT inhibitor, OPB-31121, strongly inhibited STAT3 and STAT5 phosphorylation and showed a significant antitumor effect on leukaemia with STAT-addictive oncokinases [126,127].

Oligonucleotide inhibitors of STAT3 such as siRNA and antisense RNA oligonucleotides have been investigated in order to inhibit transcriptional machinery of genes affected by JAK/STAT pathway. An example of this is use of a STAT3 decoy oligonucleotide with overall antitumor effects including inhibition of cyclin D1 and Bcl-xL transcription and angiogenesis in HNSCC both in in vitro and in vivo studies [128,129,130].

AZD9150, a synthetic antisense oligonucleotide molecule targeting STAT3 by inhibition of mRNA translation, has demonstrated antitumor activity in xenograft models. It is currently being studied in clinical trials of metastatic HNSCC as a monotherapy or combined with MED14736; an immunotherapy which blocks the interaction between PD-1 and PD-L1 (NCT02499328) [131].

Various in vitro and in vivo studies are being performed on JAK inhibitors (mostly JAK2) and many of these have reached preclinical and clinical studies. Currently, most in clinical development are orally available small molecule kinase inhibitors which act in an ATP-competitive manner [124,131,132]. Ruxolitinib (INCB018424) is an approved JAK (Jak2) inhibitor for myelofibrosis [133], with a study currently recruiting patients with HNSCC (NCT03153982). Fedratinib (SAR302503, TG101348) is a selective Jak2 inhibitor which has been studied in myelofibrosis and was approved by the FDA in 2019. In addition to myelofibrosis the effects of SAR302503 have also been investigated in solid tumours (Phase I, NCT01836705) and (Phase I, NCT01585623). Other JAK inhibitors in clinical studies are lestaurtinib (CEP-701) and XL019, while other agents are in early stages of clinical studies and development including CYT387, SB1518 and AZD1480 [124,134].

### 2.7. TP53/RB

Replacing a mutated *TP53* gene with a wild-type gene in order to restore p53 activity has been studied for decades and now is a potential approach for HNC treatment. Adenovirus vectors expressing human full length wild-type p53 have been introduced, after initial efforts in NSCLC using a retroviral vector expressing human p53 under control of an actin promoter [135]. Unlike retroviruses that are potentially oncogenic, adenovirus does not integrate the vector DNA into host cell DNA. However, limited ability to infect all tumour cells as well as induction of the host immune system after injection and the need for repetition of the therapy are disadvantages associated with this approach [136]. Gendicine (recombinant human p53 adenovirus [Ad5RSV-p53]), was approved by the China Food and Drug Administration (CFDA) in 2003 for the treatment of HNSCC [137,138,139]. Wild-type p53 gene significantly enhanced radiotherapeutic effectiveness in patients with HNSCC [138]. Infusion of rAd-p53 and chemotherapy significantly increased survival rate of patients with stage III but not stage IV oral SCC, compared with intra-arterial chemotherapy alone (ChiCTR-TRC-09000392) [140].

A Phase III clinical trial for use of Advexin (adenovirus containing *TP53*) (INGN 201, Ad5CMV-p53) versus methotrexate in treating advanced recurrent HNSCC showed that patients with wild-type p53 had better response to Advexin, whereas patients carrying mutant p53 were more responsive to methotrexate chemotherapy which indicates the potential of p53 as a biomarker to design a targeted therapy [141]. This approach has subsequently been used in a Phase II trial (NCT03544723) investigating safety and efficacy of p53 gene therapy combined with immune checkpoint inhibitors in solid tumours including HNSCC [142]. Previous Ad-p53 clinical trials appear to have been negatively impacted by the inclusion of patients with unfavourable p53 biomarker profiles and by under dosing of Ad-p53 treatment [142]. This has important implications for Ad-p53 gene therapy and future p53-targeted therapy. Although Advexin has not been approved by the FDA [143], the use of adenovirus gene therapy for treatment of HNC was approved in 2003, and Gendicine has been commercially marketed since then and used as combinational therapy mostly with radiotherapy [136,139,144].

ONYX-015 is an adenovirus with deletion of its E1B region which inactivates p53 and therefore replicates in and lyses p53-deficient cancer cells [145]. A Phase II trial of a combination of intratumoural ONYX-015 injection with cisplatin and 5-fluorouracil in patients with recurrent HNSCC (NCT00006106) was withdrawn as it showed initial objective responses, however had no more progress after 6 months, whereas all noninjected tumours treated with chemotherapy alone had progressed [146]. Other approaches including p53-reactivating small molecules [147] targeting *CDKN2A* to reactivate p16/INK4A and CDK inhibitors are under investigation [38,148].

Head and neck cancers commonly display reduced MHC expression, hence MHC class I gene therapy may be a potential therapy to induce an antitumor response either by presenting tumour antigens or on its own as an antigen. Allovectin-7^®^ is a gene therapy product that utilises a liposomal vector and encodes the class I MHC HLA-B7. A Phase II multi-institutional study of Allovectin-7 for HNC including SCC of the oral cavity or oropharynx showed that 10% of 60 HLA-B7-negative patients achieved partial response, and 23 patients had stable disease after one cycle of treatment. Responses persisted for 21 to 106 weeks [149].

### 2.8. HPV-Mediated Pathogenesis

Human papillomavirus (HPV) infection plays a critical role in a subset of HNSCC including the oropharynx (OPSCCs). HPV genome integration in the host genome not only results in amplification of oncogenes and disruption of tumour suppressor genes, but also derives inter- and intrachromosomal rearrangements [150]. A comprehensive study showed that HPV contributes to 22% of OPSCCs and 47% of tonsillar SCCs [151,152]. Therefore, understanding the prevention and treatment strategies that reflect the mechanism of HPV infection has become a matter of importance. Currently, two types of HPV vaccines are available including preventive vaccines which are based on HPV virus-like particles, and therapeutic vaccines which are being developed to eliminate established papillomavirus infection [153]. These vaccines were initially developed for anogenital malignancy, including cervical cancer and may be used for HPV associated head and neck SCC [154,155]. Prophylactic vaccines (preventive) were developed using HPV virus-like particles (VLP) to generate neutralizing antibodies against major capsid protein, L1 and L2. In 2006, the FDA approved the quadrivalent HPV vaccine Gardasil^®^/Silgard^®^ (Merck) for the preventive control of HPV infections against four high risk HPV serotypes 6, 11, 16 and 18.

Cervarix^®^ (GlaxoSmithKline) is a human papillomavirus bivalent vaccine which was approved by the FDA in 2009 and generates cellular immunity against HPV16/18 L1 protein [156]. It has also shown partial cross-protection against HPV types 31 and 45 as well [157,158]. Although, Gardasil and Cervarix have demonstrated excellent safety and efficacy profiles (up to 80% of all cervical cancers) [159,160], prophylactic HPV vaccines do not have therapeutic effects against existing HPV infections and HPV-associated lesions [160]. In addition to prevention, targeting HPV E6/E7 and induction of cell-mediated immunity response using live viral or bacterial peptide and nucleic acid vaccines may find a use in HPV associated HNSCC. Therapeutic vaccines targeting either HPV16 E6 or E7 have shown positive results in cervical cancer [161,162,163]. Simultaneous utilisation of HPV 16 genes, L1, E6, and E7 in combination therapy with archaeosomes can be an excellent strategy to stimulate immune responses against existing HPV16-associated malignancies, holding great promise for therapeutic vaccine development [164].

A Phase I clinical trial of E6 or E7 vaccine therapy in treating advanced or recurrent cancers including HNC has recently been completed (NCT00019110). A Phase I safety study of HPV E7 DNA vaccine to treat HNC patients is currently recruiting patients (NCT01493154). A Phase II trial of HPV16 synthetic long peptide (HPV16-SLP) vaccination therapy for advanced or recurrent HPV16-induced gynaecological carcinoma, was well tolerated by patients and induced a broad IFN-γ-associated T-cell response. It was subsequently suggested to use this vaccine in combination with chemotherapy and immunomodulation [165]. Further studies for developing therapeutic vaccines to treat HPV16-induced malignancies are in progress [166,167].

ADX11-001 (Lovaxin-C) is a recombinant live-attenuated *Listeria monocytogenes* (Lm) that secretes the antigen HPV16 E7 fused to a nonhemolytic listeriolysin O protein. In a Phase I study, ADX11-001 was administered to 15 women with advanced cervical cancer and only flulike symptoms including fever and hypotension were observed [168]. A Phase II clinical trial of ADX11-001 for treatment of cervical cancer met the protocol specified benchmark for activity required and will be investigated further [169].

### 2.9. Cell Cycle Pathway

Targeting abrogated cell cycle control is a promising approach for cancer therapy particularly for HNSCC [170,171].

Carboplatin (Paraplatin) and cisplatin are the primary platinum agents used in the treatment of HNC. They interact with DNA nucleophilic groups such as GC-rich sites and form intra- and interstrand DNA and DNA-protein cross-links. These bifunctional covalent links interfere with DNA synthesis in the S phase of the cell cycle and confer tumouricidal effects [172]. Both cisplatin and carboplatin based regimens show 10% to 20% overall survival benefit about 3 years [173]. However, carboplatin shows more stability and less toxicity compared to cisplatin and consequently, carboplatin-based therapy is frequently administered to patients who are unable to tolerate cisplatin [173,174].

A phase III trial of 442 patients showed that combination of platinum-based chemotherapy (carboplatin/cisplatin) with cetuximab improved overall survival compared to fluorouracil combination in recurrent or metastatic HNSCC [64,175]. Additionally, in a multicentre, noninterventional trial, patients treated for recurrent or metastatic HNSCC either with platinum-based chemotherapy and cetuximab or radiotherapy and cetuximab showed lower tumour burden in responders [176]. Recently a Phase III trial to investigate the efficacy of adding carboplatin/5FU to Erbitux-radiotherapy in patients with locally advanced HNC was completed (NCT00609284), while more recently, a phase II trial evaluating the efficacy of carboplatin in combination with palbociclib for the treatment of unresectable recurrent or metastatic HNSCC was completed (NCT03194373). Currently, multiple trials are recruiting patients to perform comparative studies combining immunotherapy drugs (e.g., pembrolizumab), 5FU and radiation in HNSCC (NCT04428333, NCT03070366, NCT04671667).

Paclitaxel is one of a new class of agents known as taxanes, a natural product from the bark of the western yew tree. Paclitaxel stabilises microtubule polymerisation and arrests the cell cycle in mitosis. The prolonged activation of the mitotic checkpoint triggers apoptosis or reversion to the G0-phase of the cell cycle without cell division [177].

Phase II studies evaluating the efficacy and safety of weekly paclitaxel treatment in patients with recurrent or metastatic HNSCC showed promising activity with acceptable toxicity. The overall response rate was 29% according to RECIST (Response Evaluation Criteria in Solid Tumours). The median duration of response, median time to progression, and median survival time were 7.4, 3.4, and 14.3 months, respectively [178]. Currently multiple phase II-IIII trials are recruiting HNSCC patients for combination of paclitaxel with other drugs including camrelizumab (an anti-PD-1 immune checkpoint inhibitor) and cisplatin (Phase II, NCT04826679), pembrolizumab plus carboplatin (phase IIII, NCT04489888) and bleomycin and cisplatin or carboplatin (phase II, NCT03830385).

### 2.10. DNA Repair Pathway

DNA damage response pathways are activated following endogenous and exogenous damage of DNA [179]. Poly (ADP-ribose) polymerase 1 (PARP1) is a member of poly (ADP) ribose moiety enzyme superfamily which repairs single-strand and double-strand breaks [180]. Tumour cells use PARP to repair platinum-induced DNA damage and thus escape apoptosis. The activity of PARP inhibitors is based on the model of synthetic lethality, where an underlying homologous recombination repair deficiency (HRD) in tumour cells makes cells highly susceptible to PARP inhibition. The antitumor effects of PARP inhibitors are not dependent on a direct interaction with a mutated gene/protein, but instead on an underlying defect in the DNA damage repair mechanism of cancer cells themselves. Therefore, combination of platinum drugs and PARP inhibitors may block cancer cells from repairing damaged DNA ultimately leading to cellular apoptosis [181].

Olaparib (Lynparza) is a PARP inhibitor that acts against *BRCA1* or *BRCA2* mutations [182]. Olaparib received European Medicines Agency (EMA) and Food and Drug Administration (FDA) approval in 2014 for treatment of germline BRCA mutated (gBRCAm) advanced ovarian cancer with three or more prior lines of chemotherapy, gBRCAm metastatic breast cancer in 2018 and gBRCAm metastatic pancreatic adenocarcinoma in 2019. Olaparib in combination with temozolomide demonstrated substantial clinical activity in relapsed small-cell lung cancer [183].

Moreover, increasing evidence from trials in solid tumours suggests that DNA damage repair (DDR) alterations may predict response to immunotherapy [184]. Recently Psyrri et al., showed that changes in DDR signals are implicated in the response to HNSCC chemotherapy and can be exploited as novel therapeutic targets and sensitive/effective non-invasive biomarkers [179]. A Phase I trial of olaparib at 25 mg orally twice daily with concurrent cetuximab and radiation for heavy smoker patients with locally advanced HNC was well tolerated with reduced dermatitis within the radiation field. Two-year OS, PFS, local control, and distant control rates were 72%, 63%, 72%, and 79%, respectively [185]. Currently the combination of olaparib with cisplatin and durvalumab (Phase II, NCT02882308), or with pembrolizumab and carboplatin (Phase II, NCT04643379) are under investigation in patients with recurrent or metastatic HNSCC.

Other molecular targets including the Src family kinase (Dasatinib, SRC inhibitor, NCT00882583) and Cyclin Dependent Kinase complex (CDK) (pablociclib, selective CDK 4/6 Inhibitors, NCT03024489) are currently being investigated in combination with cetuximab and radiotherapy in locally advanced HNC patients.

### 2.11. Hypoxia and Angiogenesis

Cells under hypoxic conditions are known to be resistant to most anticancer drugs for several reasons [186] including their distant location from blood vessels which also result in decreased cell proliferation [187,188], increased survival of cells with loss of p53-mediated apoptosis sensitivity [189], and upregulation of genes involved in drug resistance such as genes encoding P-glycoprotein (a cell membrane protein that actively pumps many drugs out of the cell) [190,191,192]. Additionally, oxygen is required for effective action of some anticancer agents such as bleomycin [193], as well as tumour responsiveness to radiotherapy by stabilising free radicals produced by ionising radiation that causes DNA damage and cell death [194].

Radiosensitisation using hypoxic modifiers such as nitroimidazoles (5-nitroimidazole) have reached Phase I and II clinical evaluations. Among more than 7000 patients who have been studied in 50 randomized trials, treatment mostly benefitted head and neck lesions and to a lesser extent bladder tumours [195]. Radiopharmaceuticals including [(18)F]EF3 which is a labelled 2-nitroimidazole hypoxia marker [196], fluorodeoxyglucose ([18F]FDG, 18F-FDG or FDG) [197], fluoromisonidazole (F-FMISO) and F-fluoroazomycin-arabinoside (F-FAZA) [198] have been used for visualising hypoxia using positron emission tomography (PET) in head and neck tumours. The effect of other agents to improve HNC oxygenation have been studied in clinical trials including hyperbaric oxygen (HBO) (100% oxygen) [199,200,201,202,203,204], carbogen (95% oxygen plus 5% carbon dioxide) and nicotinamide [205,206], and hypoxic cytotoxin tirapazamine [207,208]. In addition to many studies that have been conducted to identify hypoxia gene expression signatures in HNC, reviewed by Toustrup et al. [209], they developed a 15-gene hypoxia gene expression classifier with prognostic and predictive impact for hypoxia-modifying therapy with nimorazole in conjunction with radiotherapy [210]. Furthermore, a 26-gene hypoxia signature showed benefit from hypoxia-modifying treatment including carbogen and nicotinamide in combination with accelerated radiotherapy in laryngeal cancer and is to be evaluated in a clinical trial [211].

To date, there are two main approaches available for targeting angiogenesis including targeting VEGF ligand and inhibition of VEGF receptor tyrosine kinase [212]. Bevacizumab (Avastin^®^) is a fully humanised IgG1 mAb that binds to all five forms of VEGF and prevents the interaction of VEGF to its receptors on endothelial cells surface. It has been FDA-approved for a variety of cancers including metastatic colorectal cancer (first line therapy, 2004/second line therapy, 2006), advanced nonsquamous non-small-cell lung cancer in combination with carboplatin/paclitaxel chemotherapy (first line therapy, 2006), glioblastoma (second line therapy, 2009), metastatic RCC (2009), metastatic HER2-negative breast cancer (2011), and metastatic colorectal cancer in combination with fluoropyrimidine-based chemotherapy (2013). A Phase III clinical trial for adding bevacizumab to chemotherapy for treatment of patients with recurrent or metastatic HNC (NCT00588770) did not improve OS but improved the response rate and PFS with increased toxicities. Median OS was 12.6 months with bevacizumab plus chemotherapy (BC) and 11.0 months with chemotherapy alone. At 2, 3, and 4 years, the OS rates were 25.2% v 18.1%, 16.4% v 10.0%, and 11.8% v 6.4% for BC versus chemotherapy, respectively [213].

Bevacizumab has reached Phase II trials in combination with other therapies including erlotinib [214], pemetrexed [215], cetuximab [216] and cisplatin and intensity-modulated radiation therapy for Stage III or Stage IV HNC (NCT00423930) [217]. Although, it has been proven to be effective, it has been associated with serious bleeding as a side effect [218].

Small molecules that inhibit VGFR are mostly multikinase inhibitors, tyrosine kinase inhibitors (TKI), including sorafenib, vatalanib, and sunitinib. Sorafenib is a Raf inhibitor which also inhibits VEGFR2, VGFR3 and PDGFR [219]. It has been used as a single agent [99] and is in active clinical trials in combination with other therapies such as carboplatin and paclitaxel (Phase II, NCT00494182) [220], cetuximab (Phase II, NCT00939627) [221] and cisplatin and docetaxel (Phase I/II, NCT02035527) [222] in HNSCC patients.

Sunitinib malate (Sutent^®^) is also a multikinase inhibitor which inhibits VGFR1, VGFR2, VGFR3, PDGFR, RET and c-KIT receptors [223]. It is an approved FDA agent for conditions including gastrointestinal stromal tumour (2006), advanced (metastatic) RCC (2006) and pancreatic neuroendocrine tumours (2011). Although, it has been well tolerated as a single agent therapy in HNSCC patients [224], it has been associated with significant bleeding side effect [225]. Currently, a Phase IB trial of sunitinib in combination with radiation therapy for HNC was recently completed (NCT00437372).

Vandetanib is a dual tyrosine kinase inhibitor that inhibits both EGFR and VEGFR simultaneously [226]. It was approved by FDA for the treatment of symptomatic or progressive medullary thyroid cancer in patients with unresectable, locally advanced, or metastatic disease in 2011. Sano et al. showed that adding vandetanib to cisplatin, and radiation could overcome cisplatin and radioresistance in in vitro and in vivo HNSCC models [54]. Vandetanib in combination with cisplatin and radiation (Phase II, NCT00720083) was withdrawn, while a Phase II clinical trial on preventing cancer in patients with precancerous head and neck lesions was recently completed (NCT01414426).

Vatalanib (PTK787/ZK-222584) is a small molecule that inhibits VGFR, EGFR, PDGFR, RET and c-KIT receptors and is under investigation for the treatment of solid tumours [227]. Vatalanib has successfully passed Phase II clinical trial as an oral angiogenesis inhibitor in patients with stage IIIB/IV NSCLC [228]. In vitro studies using HNC tumour cell lines showed that vatalanib could block VEGF downstream targets including Bcl-2 and CXCL8 expression in endothelial cells [229]. The Phase I study of vatalanib in combination with everolimus for advanced solid tumours (NCT00655655) was recently completed.

A Phase II trial of axitinib demonstrated a low objective response rate but favourable disease control rate of 77% and median OS of 10.9 months with an acceptable toxicity profile [230].

Other VEGFR inhibitors in clinical trials in HNC include cediranib (AZD-2171, recentin) (Phase II, NCT00458978) and pazopanib as monotherapy (Phase II, NCT01377298) and in combination with cetuximab (Phase I, NCT01716416), and nilotinib (Phase I, NCT01871311).

### 2.12. Host Immunity

Cancer immunotherapy utilizes the immune system to attack tumours. Immune checkpoint inhibitors provide a natural brake on the immune system so that immune cells (T cells) can recognize and target cancer cells. Head and neck cancers are immunosuppressive and therefore likely to respond well to immunotherapy [231]. The programmed cell death 1/programmed cell death ligand 1 (PD-1/PD-L1) and CTLA-4 (cytotoxic T-lymphocyte-associated protein 4) are critical targets for immune checkpoint therapies.

Antibodies targeting the checkpoint molecules including PD-1, PD-L1 and CTLA-4 have resulted in durable responses and improved survival in patients with advanced cancer. Ipilimumab (Yervoy), an anti-CTLA-4 monoclonal antibody (human IgG1 k), targets CD28-CD80/CD86 co-stimulatory pathways and is the only CTLA-4 inhibitor to receive FDA approval in 2011 [232,233]. Tremelimumab, a human IgG2 anti-CTLA-4 antibody, has undergone various clinical trials for HNSCC (NCT02369874, NCT02551159, NCT02319044) [234,235,236].

In addition to ipilimumab, there are six approved monoclonal antibodies targeting PD-1 and PD-L1, namely, nivolumab (Opdivo), pembrolizumab (Keytruda), avelumab (Bavencio), atezolizumab (Tecentriq) durvalumab (Imfinzi) and cemiplimab (Libtayo). The first two are anti-PD-1 antibodies, and the latter four are anti-PD-L1 antibodies (Table 2). Nivolumab and pembrolizumab have been approved by the FDA for treatment of patients with HNSCC with relapse or metastasis who demonstrate cisplatin resistance [237].

Nivolumab, an anti-PD-1 monoclonal antibody, was the first immunotherapy approved by the FDA for the treatment of HNSCC following a Phase III clinical trial (Checkmate 141, NCT02105636) in 2016 [238]. Nivolumab treatment of recurrent and metastatic HNSCC has increased the overall survival time and disease-free survival regardless of the expression levels of PD-L1 or the p16 status of the cancer. Median OS was 7.5 months in the nivolumab group versus 5.1 months in the group that received standard therapy. OS was significantly longer with nivolumab than with standard therapy and the estimates of the 1-year survival rate were approximately 19 percentage points higher with nivolumab than with standard therapy (36.0% vs. 16.6%). Median PFS was 2.0 months with nivolumab versus 2.3 months with standard therapy. The rate of PFS at 6 months was 19.7% with nivolumab versus 9.9% with standard therapy. The response rate was 13.3% in the nivolumab group versus 5.8% in the standard-therapy group [238,239]. The combination of nivolumab and ipilimumab has shown effective results in melanoma [240,241] and RCC [242]. The combination is under investigation in other solid tumours including HNSCC.

Pembrolizumab, a humanised IgG4-κ monoclonal antibody targeting PD-1, was first approved by the FDA in 2017 for any unresectable or metastatic solid tumour with certain genetic anomalies such as mismatch repair deficiency or microsatellite instability [243]. A Phase III study of pembrolizumab alone or with chemotherapy versus cetuximab with chemotherapy for recurrent or metastatic HNSCC showed that pembrolizumab plus platinum and 5-fluorouracil is an appropriate first-line treatment for recurrent or metastatic HNSCC, and pembrolizumab monotherapy is an appropriate first-line treatment for PD-L1-positive recurrent or metastatic HNSCC [55]. Pembrolizumab showed significant treatment results in the treatment of relapsed or metastatic HNSCC in the Phase III clinical study (KEYNOTE 048; NCT02358031) in 2019. The combination of pembrolizumab and chemotherapy improved the OS to 13.0 months from 10.7 months for the combination of cetuximab and chemotherapy. Based on the observed efficacy and safety results, pembrolizumab and chemotherapy are now first-line treatment for patients with recurrent or metastatic HNSCC, whereas pembrolizumab monotherapy is the first-line treatment for patients with relapsed or metastatic PD-L1-positive HNSCC [55]. Other anti-PD-1 Phase II/III trials currently underway including NCT03813836 (pembrolizumab in recurrent/metastatic HNSCC with WHO performance status 2), NCT02521870 (pembrolizumab plus intratumoral SD-101 in anti-PD-1/PD-L1 treatment-naïve recurrent/metastatic HNSCC), NCT02741570 (nivolumab plus ipilimumab vs. the EXTREME regimen as first-line treatment in recurrent/metastatic HNSCC), NCT03040999 (pembrolizumab plus cisplatin plus RT vs. cisplatin plus RT alone in locally advanced HNC), and NCT02707588 (pembrolizumab plus RT vs. cetuximab plus RT in locally advanced HNC).

A Phase I immuno-radiotherapy with cetuximab and avelumab (Bavencio), a PD-L1 inhibitor, showed that cetuximab-RT plus avelumab is feasible in patients with advanced-stage HNSCC who are not good candidates for cisplatin treatment. Tumour recurrence was 50% after a median of 12 (8–26) months follow-up [244]. Patients are currently being recruited in a Phase I clinical trial to assess the feasibility of intratumoural administration of ipilimumab monotherapy prior to surgical resection, and to assess the immune system response to treatment (NCT02812524). Currently there are multiple Phase I/II clinical trials recruiting patients for combinational therapies, including Phase I/II trial of durvalumab, tremelimumab and radiation therapy in recurrent and metastatic HNSCC (NCT03522584), Phase II of nivolumab and ipilimumab for recurrent and metastatic HNSCC (NCT03620123), and Phase I of Interleukin-15 Superagonist (N-803) and ipilimumab in patients with advanced head and neck cancer (NCT04290546). This is in addition to a Phase III trial assessing atezolizumab after definitive local therapy in high-risk locally advanced HNC (NCT03452137).

## 3. Combination Therapies

It is clear from this paper and from many others detailing currently useful and agreed protocols for managing HNSCC, that a combination of treatment modalities and a combination of pharmacotherapeutics are required to manage and treat HNSCC effectively. Assessment of clinical trials registered with ClinicalTrials.gov shows that about 30% are assessing chemotherapeutic approaches, 15% immunotherapies, 15% targeted therapies, 10% radiotherapies, and 5% surgical approaches, with about another 15% assessing combination therapies. These combination therapies cross the traditional radiation oncology/medical oncology/surgery divides, and are aimed at maximizing the benefits of one treatment modality and limiting the adverse effects of another. These apply equally to radiation-based combinations, neoadjuvant chemotherapy, and adjuvant therapy, with a significant current focus on recurrent and metastatic disease. This is seen in routine oncological practice worldwide. Moving forward however, combination molecular therapies will become more common, as our understanding of the molecular and signalling pathways of HNSCC as a heterogeneous group of diseases increases. These can be applied to the same clinical settings and scenarios, but potentially as first line therapies.

Current treatment approaches are still very toxic, highlighting the need for more targeted approaches, stratification of tumour types and substratification of applicable and druggable biomarkers to reduce toxicity and improve outcomes. Further understanding of the tumour microenvironment and tumour immunology would allow better use of immunotherapies tailored appropriately to the patient and their tumour. Further exploration of the role of HPV and the oral microbiome in initiation of HNSCC is equally important as that may dictate combinations of molecular therapies with greater utility, depending on etiology, keratinocyte differentiation, pathogenesis and potential response to therapy. This may underpin the rationale for combination therapies where the input of the immune system and the influence of the microbiome are taken into account with the choice of PD-1/PD-L1 immunotherapies coupled with PI3K/AKT/mTOR inhibitors. An equally attractive approach is based on the activity of PARP inhibitors and radiation therapy, or PARP inhibitors and immunotherapies. Equally, combination approaches in HPV-negative tumours could make simultaneous use of EGFR inhibitors (Cetuximab), MET inhibitors (Ficlatuzumab) and p53-targetted therapies be it adenoviral p53 gene therapy or use of small molecules to restore *TP53* function or disrupt inactivation of wild-type p53. Regardless of the combinations to be tested or trialled, the rationale for their combined use will depend on the overall molecular profile of the patient’s tumour gleaned through a precision medicine approach.

## 4. Conclusions and Future Directions

Currently, only cetuximab, pembrolizumab and nivolumab are approved by the FDA as molecular therapies for HNSCC. All three drugs are only indicated for locally advanced, recurrent or metastatic disease, and in combination with radiotherapy or other chemotherapeutic agents such as cisplatin or 5-fluorouracil. Surgery continues to be first line therapy for oral cavity SCC, with chemoradiotherapy indicated for oropharyngeal SCC. Although significant advances have been made in our understanding of the genes and associated signalling pathways involved in HNC, successful molecular therapies have not remarkably changed clinical practice as first line therapy. Molecular approaches however continue to serve patients with recalcitrant disease, or with particular mutational or immune profiles such as that seen in application of immune checkpoint inhibitors. More novel approaches with agents taking advantage of synthetic lethality or those exploiting p53 mutations utilising gene therapy hold hope for translation into clinical practice. While vaccination against oncogenic subtypes of HPV hold promise for a reduction in oropharyngeal and other HPV-induced HNCs, there remains a significant burden of disease that is nonresponsive to currently accepted standard therapies, and for which molecular therapies may still hold significant advantage. Taking full advantage of precision medicine in this regard is underpinned by our ability to accurately identify individual patient genomic variations and to tailor targeted therapy based on this. Incorporating screening for actionable genomic targets and treating with a matched targeted therapy in future clinical trials is but one approach to consider if there are to be significant benefits for patients moving forward. Design of new biomarker-driven clinical trials for HNSCC is urgently required if we are to successfully incorporate molecular therapies as standard treatment for head and neck cancers.

## Figures and Tables

**Table 1:** Selected drugs and compounds currently available for treatment of head and neck squamous cell carcinoma.

Drug (Brand Name)	Mechanism of Action	FDA Approved for Head and Neck Cancer	FDA Approval Year/Progression Phase	Key ClinicalTrials.gov Identifiers
Docetaxel (Taxotere)	Microtubule stimulant	Yes	2006	NCT00003888
Cetuximab (Erbitux)	EGFR mAb	Yes	2006	NCT00004227
Cisplatin in combination with 5- Fluorouracil and Cetuximab	DNA synthesis and mRNA transcription inhibitor with EGFR mAb	Yes	2011	NCT00122460
Pembrolizumab (Keytruda)	PD-1 inhibitor	Yes	2017	NCT02358031 NCT02252042
Nivolumab (Opdivo)	PD-1 inhibitor	Yes	2016	NCT02105636
Zalutumumab (HuMax-EGFR)	EGFR mAb	-	Phase III	NCT00382031
Panitumumab (Vectibix)	EGFR mAb	-	Phase III	NCT00460265 NCT00820248
Nimotuzumab	EGFR mAb	-	Phase III	NCT00957086
Gefitinib (Iressa)	EGFR TKI	-	Phase III	NCT00088907 NCT00206219
Erlotinib (Tarceva)	EGFR TKI	-	Phase II	NCT01515137
Lapatinib (Tykerb/Tyverb)	EGFR/HER2 TKI	-	Phase II	NCT00098631 NCT01044433 NCT00424255
Foretinib	c-MET and VEGFR-2 inhibitor	-	Phase II	NCT00725764
Bevacizumab (Avastin)	Anti-VEGF mAb	-	Phase III	NCT00588770
Sunitinib (Sutent)	Multi-target TKI (PDGFR, VEGFR, c-KIT)	-	Phase I	NCT00437372
Sorafenib (Nexavar)	Multi-target TKI (VEGFR, PDGFR, RAF)	-	Phase II	NCT00096512 NCT00494182 NCT00939627
Buparlisib aka BKM120	PI3K inhibitor	-	Phase II	NCT01527877 NCT01737450 NCT01816984 NCT02113878
Alpelisib aka BYL719	PI3K inhibitor	-	Phase I	NCT02282371
Sonolisib aka PX-866	PI3K inhibitor	-	Phase II	NCT01252628 NCT01204099
Temsirolimus (Torisel)	mTOR kinase inhibitor	-	Phase II	NCT01172769 NCT01256385 NCT01016769
Everolimus (Afinitor)	mTOR kinase inhibitor	-	Phase II	NCT00942734
Tipifarnib (Zarnestra)	RAS inhibitor	-	Phase II	NCT02383927 NCT03719690
Ad5RSV-p53 (Gendicine)	p53 stimulant	-	Phase II/III	-
Ad5CMV-p53 (Advexin)	p53 stimulant	-	Phase II/III	NCT00041613 NCT00041626 NCT03544723
Lontucirev aka ONYX-015	p53 stimulant	-	Phase II	NCT00006106
Olaparib (Lynparza)	PARP inhibitor	-	Phase II	NCT02882308 NCT04643379

mAB: monoclonal antibody. TKI: tyrosine kinase inhibitor. FDA: U.S Food and Drug Administration.

**Table 2:** Immune checkpoint inhibitors.

Drug (Brand Name)	Target	Class	First FDA Approval	Cancer Type
Nivolumab (Opdivo)	PD-1	IgG4	2014	melanoma, lung cancer, renal cell carcinoma (RCC), Hodgkin lymphoma, head and neck cancer (HNC), colon cancer, and liver cancer
Pembrolizumab (Keytruda)	PD-1	IgG4-κ	2017	melanoma, lung cancer, head and neck cancer (HNC), Hodgkin lymphoma, stomach cancer and colorectal cancer
Atezolizumab (Tecentriq)	PD-L1	IgG1	2016	urothelial carcinoma, non-small-cell lung cancer (NSCLC), triple-negative breast cancer (TNBC), small cell lung cancer (SCLC), and hepatocellular carcinoma (HCC)
Avelumab (Bavencio)	PD-L1	IgG1	2017	Merkel-cell carcinoma (MCC)
Durvalumab (Imfinzi)	PD-L1	IgG1	2017	bladder and lung cancer
Cemiplimab (Libtayo)	PD-L1	IgG1	2018	squamous cell skin cancer
Ipilimumab (Yervoy)	CTLA-4	IgG1	2011	melanoma, RCC, dMMR metastatic colorectal cancer
